# Preparation of a Magnetic Ti-IMAC Material Based on Thiol-Ene Click Reaction and the Application in Intact Phosphoprotein Enrichment

**DOI:** 10.3390/molecules31030396

**Published:** 2026-01-23

**Authors:** Yan Lu, Sen Zhang, Hong-Yan Ge, Han-Yue Yang, Feng Zhang, Yi-Fan Pan, Hong-Zhen Lian

**Affiliations:** 1 State Key Laboratory of Analytical Chemistry for Life Science, Jiangsu Key Laboratory of Clean Energy Catalysis and Intelligent Green Chemical Engineering, School of Chemistry & Chemical Engineering and Center of Materials Analysis, Nanjing University, Nanjing 210023, China; ly181850106@163.com (Y.L.);; 2 Jiangsu Deyuan Pharmaceutical Co., Ltd., 21 Jinqiao Road, Lianyungang 222002, China

**Keywords:** phosphoprotein, intact protein, immobilized metal ion affinity chromatography, magnetic solid phase extraction, MALDI-TOF MS, enrichment

## Abstract

Protein phosphorylation is a crucial post-translational modification that regulates protein activity, cellular signaling, transcriptional regulation, and cell cycle control. However, the analysis of phosphoproteins in biological samples is often compromised by complex sample matrices and interference from high-abundance proteins. While the top-down phosphoproteomics strategy enables comprehensive analysis of post-translational modifications based on intact proteins, its requirement for higher protein purity due to low protein ionization efficiency poses stern challenges. Consequently, developing appropriate enrichment methods for phosphoproteins in practical samples becomes essential. Immobilized metal ion affinity chromatography (IMAC) represents a common strategy for phosphorylated protein separation and enrichment. Among metal ions, Ti^4+^ has gained widespread application as IMAC chelating ligands due to its capacity to form multiple coordination networks and its high selectivity for phosphorylated protein enrichment, leveraging the strong chelating ability of phosphate groups toward metal ions. This paper presents the design and preparation of a novel magnetic Ti-IMAC nanocomposite, MNP@MPTMS–VPA–Ti(IV). The material is modified with phosphate groups via facile thiol-ene click chemistry and then immobilizes Ti^4+^, enabling selective enrichment of intact phosphoproteins through IMAC affinity. The efficiency of enrichment was evaluated using subsequent matrix-assisted laser desorption/ionization time-of-flight mass spectrometry (MALDI-TOF MS) for detection and analysis. This Ti-IMAC material-based magnetic solid-phase extraction (MSPE)-MALDI-TOF MS protocol has been successfully applied to enrich intact phosphoproteins in milk and eel mucus with high selectivity, sensitivity, and suitability.

## 1. Introduction

As a crucial post-translational modification (PTM), protein phosphorylation plays significant biological roles through regulating protein activity, mediating cellular signal transduction, participating in gene expression regulation, and controlling cell cycle progression [1,2,3]. In-depth research on phosphoproteins not only facilitates the discovery of disease-specific biomarkers but also provides critical foundations for clinical diagnosis and therapeutic evaluation [4,5,6]. However, technical challenges, such as the low abundance of phosphoproteins and complex background interference in real samples, make direct analysis by conventional mass spectrometry difficult. Therefore, developing novel materials and detection methods for efficient enrichment of intact phosphoproteins is essential.

To date, numerous strategies have been developed for phosphoprotein separation and enrichment, including chemical modification methods [7,8], immunoprecipitation [9], strong cation/anion exchange chromatography (SCX/SAX) [10], metal oxide affinity chromatography (MOAC) [11,12], and immobilized metal ion affinity chromatography (IMAC) [13,14]. Combined with magnetic solid-phase extraction technology (MSPE), IMAC affinity materials have attracted extensive research due to their excellent enrichment performance, low cost, operational simplicity, and broad applicability [15]. The enrichment mechanism of IMAC primarily relies on the affinity between positively charged metal ions and phosphate groups. The core principle involves immobilizing high-valence metal ions (Fe^3+^, Ti^4+^, Ga^3+^, etc.) through chelating ligands on substrate surfaces, such as iminodiacetic acid (IDA) and nitrilotriacetic acid (NTA) [16,17], where the unsaturated coordination orbitals of metal ions form stable coordination bonds with phosphate groups in phosphoproteins/peptides, enabling selective enrichment. Thus, the appropriate selection of chelating ligands and metal ions is crucial for enhancing IMAC material adsorption efficiency.

In the recent two decades, research in IMAC has focused on high-valence metal ions (Zr^4+^, Sn^4+^, and Ti^4+^), with some rare-earth ions (Gd^3+^, La^4+^, and Ce^4+^) also being applied, albeit with cost concerns [18,19]. Different metal ions exhibit varying phosphoprotein enrichment efficiencies, with Ti^4+^ demonstrating superior capture performance through enhanced selectivity and sensitivity [20]. The hexacoordinate nature of Ti^4+^ enables simultaneous multi-phosphate coordination, forming extensive coordination networks. This characteristic not only strengthens binding capacity for mono-phosphorylated sites but also effectively captures poly-phosphorylated proteins, overcoming traditional IMAC limitations in recognizing complex modification patterns [21]. Compared with conventional IDA and NTA ligands, phosphate groups have been widely adopted as IMAC modifiers due to their inherent similarity to phosphorylation modifications and excellent metal-ion chelation capabilities [22,23,24]. There are numerous methods for grafting phosphate groups [23,25,26,27]. For example, in a traditional Ti-IMAC material Zhou et al. prepared [27], the graft of phosphate groups was based on the Eschweiler–Clarke reaction, which requires high acidity and elevated temperatures, putting forward a higher requirement for the stability of the substrate of materials. Liu et al. [28] recently reported a newly designed IMAC adsorbent composite constructed on the graphene matrix coated with mesoporous silica. The work employed free radical polymerization for the grafting of phosphate groups, but it required a long-term, high-temperature reaction and a large amount of organic solvents. Click chemistry has gained significant attention since its introduction by Sharpless in 2001 [29] and was awarded the 2022 Nobel Prize in Chemistry. These reactions, such as copper-catalyzed azide-alkyne cycloaddition (CuAAC) [30], strain-promoted azide-alkyne cycloaddition (SPAAC) [31], sulfur(VI) fluoride exchange (SuFEx) [32], and thiol-ene click reactions [33], are highly selective and orthogonal, which do not or barely yield side products. Compared with other click reactions, thiol-ene click reactions proceed under milder conditions with easily available materials, require no metal catalysts, and are particularly suitable for oxygen/moisture-sensitive biological systems or functional material synthesis [34]. Therefore, it can serve as a milder way to prepare nanocomposites for phosphoproteomics analysis from our perspective.

Since the development of electrospray ionization (ESI) [35] and matrix-assisted laser desorption/ionization (MALDI) [36,37], proteomics based on mass spectrometry have a widespread application. Two common strategies, bottom-up and top-down, are extensively employed in MS-based proteomics. In recent years, phosphoproteomics research has predominantly relied on the bottom-up strategy [38,39,40,41], which involves detection and analysis at the peptide level followed by bioinformatics-based reconstruction of protein primary structures. Recent Ti-IMAC materials mentioned before [22,23,28] for phosphoproteomics analysis are also mostly based on the bottom-up strategy. However, peptide-level analysis presents certain limitations, primarily including the loss of post-translational modification information and peptide assignment ambiguities. During enzymatic digestion, shorter peptides that cannot be detected by mass spectrometry may form [42,43]. Additionally, post-translational modifications of proteins may affect enzymatic digestion efficiency [44], ultimately leading to incomplete interpretation of protein post-translational modification information. Furthermore, for homologous proteins with similar structures, shared peptides may exist after enzymatic digestion, making the assignment of such peptides challenging in complex samples [45]. Top-down strategy-based proteomics can more comprehensively acquire post-translational modification information of proteins. However, due to the low ionization efficiency of intact proteins, this strategy imposes higher requirements on sample purity and mass spectrometry resolution. Therefore, proper separation and enrichment methods for intact proteins are crucial for advancing top-down proteomics.

In this study, a magnetic Ti-IMAC nanocomposite (MNP@MPTMS-VPA-Ti(IV)) was prepared based on thiol-ene click chemistry and applied for intact phosphoprotein enrichment for the first time. The 3-mercaptopropyltrimethoxysilane (MPTMS) silica layer of the material provides dual functionality, protective shielding for the magnetic core and thiol groups essential for thiol-ene click reactions. Phosphate group modification enhances hydrophilicity and biocompatibility, while the flexible long carbon chains formed by thiol-ene reaction partially reduce steric hindrance during macromolecular protein adsorption. The nanomaterial was applied for phosphoprotein enrichment based on IMAC interaction between Ti^4+^ and phosphoproteins. It demonstrates excellent performance in the enrichment of intact *β*-casein, with a low limit of detection and high selectivity, and has been successfully implemented for phosphoprotein separation in complex samples such as milk and skin mucus of eel.

## 2. Results and Discussion

### 2.1. Characterization of MNP@MPTMS–VPA–Ti(IV)

**Scheme 1:**
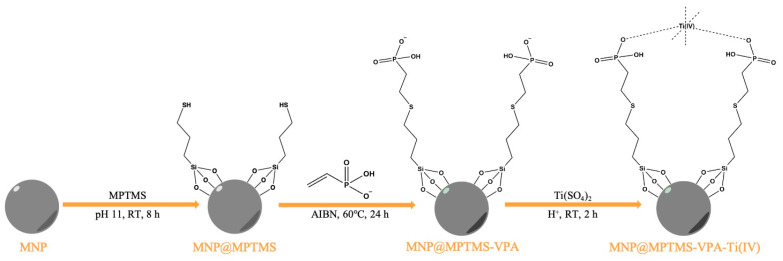
Schematic illustration of the synthesis strategy of MNP@MPTMS–VPA–Ti(IV).

The synthesis route of MNP@MPTMS–VPA–Ti(IV) is illustrated in Scheme 1. Fe_3_O_4_ nanoparticles were first prepared via the solvothermal method. The surface of the magnetic nanoparticles was coated with a silica layer using (3-mercaptopropyl)trimethoxysilane (MPTMS), which provides the thiol groups required for thiol-ene click reaction. Subsequently, vinylphosphonic acid (VPA) is used to functionalize the surface of the material with phosphate groups. Finally, Ti(IV) is immobilized onto the phosphate-modified material surface to obtain the magnetic Ti-IMAC nanomaterial.

**Figure 1:**
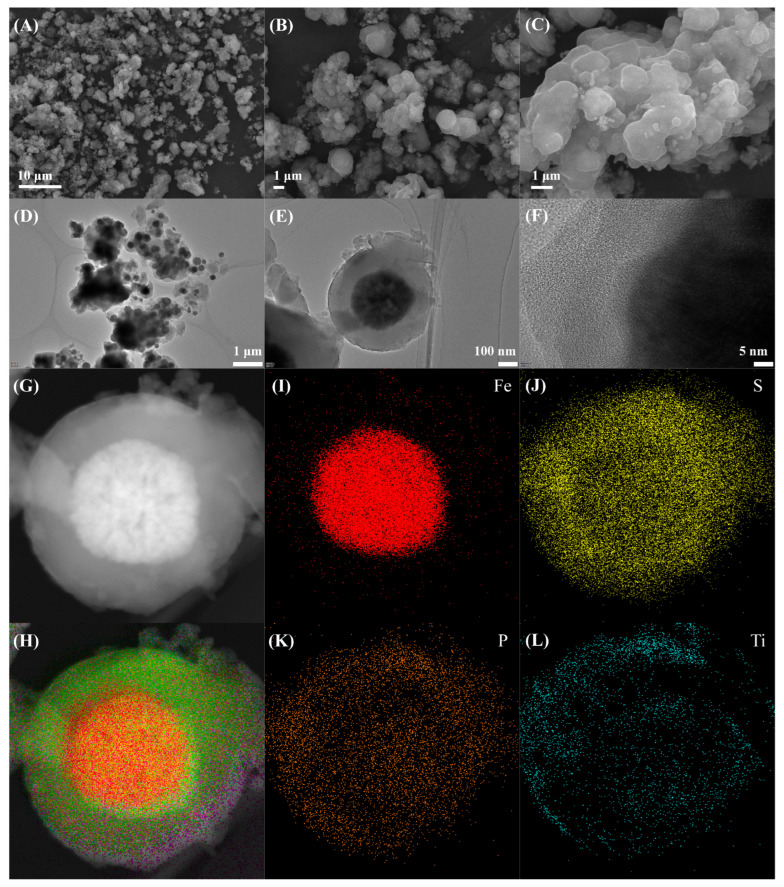
SEM images (**A**–**C**), TEM images (**D**–**F**), TEM dark field image (**G**) of MNP@MPTMS–VPA–Ti(IV) and corresponding EDS elemental mapping picture: all (**H**), Fe (**I**), S (**J**), P (**K**), and Ti (**L**).

The morphology of synthesized MNP@MPTMS–VPA–Ti(IV) was characterized using scanning electron microscopy (SEM) and transmission electron microscopy (TEM), as shown in Figure 1. TEM images indicated a distinct core–shell structure, with the magnetic core exhibiting a particle size of approximately 200–300 nm and the coating layer measuring about 100–150 nm, which was in agreement with the result in the size distribution diagram (Figure S1) from SEM images. High-resolution TEM (HRTEM) images revealed clear lattice fringes in the magnetic core region, whereas the coating layer showed no fixed crystalline structure. Energy dispersive spectroscopy (EDS) elemental mapping demonstrated homogeneous distribution of Fe, S, P, and Ti elements throughout the material.

**Figure 2:**
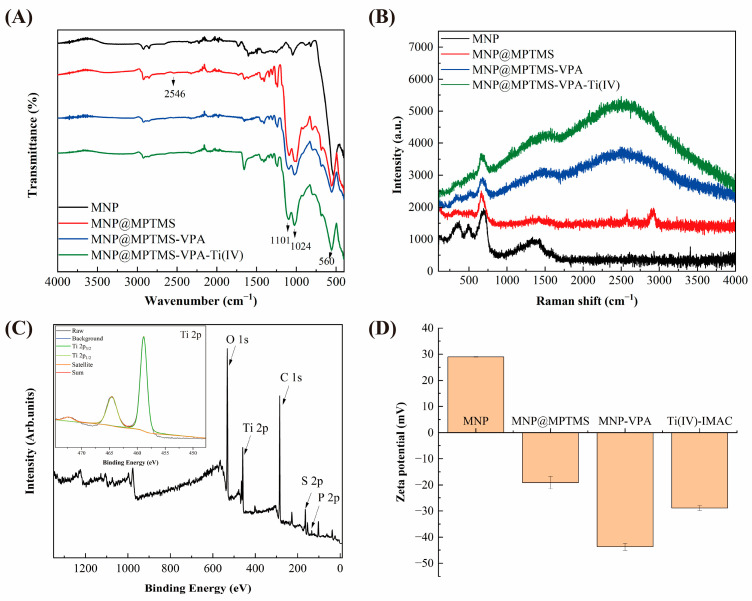
ATR-FTIR spectra (**A**) and Raman spectra (**B**) of MNP, MNP@MPTMS, MNP@MPTMS–VPA and MNP@MPTMS–VPA–Ti(IV); XPS spectra (**C**) of MNP@MPTMS–VPA–Ti(IV), with the deconvoluted segmented XPS spectra of Ti 2p inserted; Zeta potential (**D**) of MNP, MNP@MPTMS, MNP@MPTMS–VPA, and MNP@MPTMS–VPA–Ti(IV).

As shown in Figure 2A, ATR-IR was employed to characterize the functional groups of Fe_3_O_4_, MNP@MPTMS, MNP@MPTMS–VPA, and MNP@MPTMS–VPA–Ti(IV). The synthesized Fe_3_O_4_ particles exhibit an Fe–O bond absorption peak at 560 cm^−1^. After coating with mercaptosilane MPTMS, distinct Si–O and Si–O–Si absorption peaks appear at 900–1100 cm^−1^, while the peaks at 2800–2900 cm^−1^ correspond to the stretching vibration of –CH_2_–, and a weak absorption peak of mercapto (–SH) emerges at 2550–2560 cm^−1^. In the phosphate-modified material MNP@MPTMS–VPA via thiol-ene click reaction, according to the literature [23], the infrared peaks of P=O stretching vibration, P–O asymmetric stretching vibration, and P–O symmetric stretching vibration are supposed to be at 1115, 1043, and 966 cm^−1^, respectively. However, due to the presence of the silane coating, these peaks may be obscured by the strong absorption peaks of Si–O and Si–O–Si. Nevertheless, a relative weakening of the mercapto (–SH) absorption peak can be observed, indicating the occurrence of the thiol-ene click reaction. After the immobilization of Ti(IV) ion, no significant changes were observed in the spectra except a weak increase in a peak at 1600–1700 cm^−1^ corresponding to the stretching vibration of C=O. The increase may be attributed to the residual formic acid in the washing procedure. In view of relatively weak changes in the thiol absorption peaks observed in IR spectroscopy, Raman spectroscopy was employed as a complementary characterization technique for the material, as shown in Figure 2B. The three peaks at approximately 337, 489, and 687 cm^−1^ in the spectrum correspond to the characteristic Raman peaks of the magnetic nanoparticle (Fe_3_O_4_). After Fe_3_O_4_ was coated by MPTMS, a significant –CH_2_– peak emerged in the 2800–2900 cm^−1^ region, as well as the peak of –SH at 2573 cm^−1^, in the MNP@MPTMS spectrum. Furthermore, after the thiol-ene click reaction with VPA, an apparent decrease was observed in the characteristic thiol peak of the MNP@MPTMS–VPA spectrum. Collectively, the characterization results from both infrared and Raman spectroscopy preliminarily confirm the successful occurrence of the thiol-ene click reaction and the effective modification of phosphate groups.

Powder X-ray diffraction (PXRD) characterization was performed on the synthesized MNP, MNP@MPTMS, MNP@MPTMS–VPA, and MNP@MPTMS–VPA–Ti(IV), as shown in Figure S2. Comparison with standard reference cards confirmed that all materials consistently displayed the characteristic diffraction peaks of Fe_3_O_4_. The peaks observed at 2θ = 30.2, 35.5, 43.2, 53.5, 57.1, and 62.7° correspond to the (2 2 0), (3 1 1), (4 0 0), (4 2 2), (5 1 1), and (4 4 0) crystal planes of Fe_3_O_4_, respectively. These results demonstrate that the crystal structure of the material remained unchanged throughout the reaction process.

XPS characterization was employed to verify the bonding states of different elements in the material. As shown in Figure 2C, characteristic peaks of C 1s (284 eV), O 1s (532 eV), P 2p (133 eV), S 2p (163 eV), and Ti 2p (458 eV) can be observed in the spectrum. Although the Fe 2p peak is theoretically expected around 710 eV, it remained undetectable due to the thick coating layer (100–150 nm as confirmed by prior TEM analysis) exceeding the detection depth of XPS (typically <10 nm for surface-sensitive characterization) [46]. High-resolution segmented spectra of S 2p, P 2p, and Ti 2p were deconvoluted and analyzed. As shown in Figure S3, S 2p peaks at 163.27 and 164.46 eV correspond to spin-orbit split 2p_3/2_ and 2p_1/2_ levels of sulfur in C–S–C bonds from thiol-ene click reaction, while peaks at 168.39 and 169.49 eV indicate S–O bonds likely from SO_4_^2−^ introduced during titanium immobilization. P 2p binding energies at 132.98 and 133.84 eV represent the 2p_3/2_ and 2p_1/2_ doublet of PO_4_^3−^. Ti 2p deconvolution resolved peaks at 458.83 eV (2p_3/2_) and 464.54 eV (2p_1/2_), consistent with Ti(IV) [47], along with a satellite peak at 472.31 eV. Complementary EDS analysis (Figure S4) confirmed elemental composition with atomic percentages of S (7.12%), P (0.78%), and Ti (0.08%). Collectively, these results validate the presence of S, P, and Ti in characteristic chemical states, demonstrating successful stepwise functionalization during synthesis.

Zeta potential characterization was performed on the synthesized materials to investigate surface charge variations and validate successful modification at each reaction step. Results (Figure 2D) showed a zeta potential of 29 mV for Fe_3_O_4_ nanoparticles, which shifted to −19.1 mV for MNP@MPTMS due to the negatively charged thiol groups after silane coating. Following phosphate group modification via thiol-ene click reaction, MNP@MPTMS–VPA exhibited a more negative potential of −43.7 mV. After Ti^4+^ immobilization, the overall potential measured −29.1 mV (less negative than the phosphate-modified sample) rather than reverting to positive values, as zeta potential reflects total surface charge while Ti^4+^ ions distribute locally at phosphate sites. This potential shift confirms successful Ti^4+^ fixation.

Given the application as a magnetic solid-phase extraction (MSPE) adsorbent, the magnetic responsiveness of the nanomaterials was evaluated using a vibrating sample magnetometer (Figure S5). Solvothermally synthesized Fe_3_O_4_ showed a saturation magnetization of ~80 emu·g^−1^, indicating strong paramagnetic behavior. After silica coating and thiol-ene reaction, MNP@MPTMS–VPA exhibited reduced saturation magnetization (~35 emu·g^−1^) due to the thick non-magnetic coating. After the chelation of Ti^4+^, the nanocomposite maintained ~35 emu·g^−1^ magnetization and responded rapidly to external magnetic fields. These results confirm sufficient magnetic responsiveness for efficient magnetic separation in extraction applications.

N_2_ adsorption-desorption analysis (Figure S6) determined the specific surface area of MNP@MPTMS–VPA–Ti(IV) for adsorption capacity evaluation. The isotherm displayed a Type-IV curve, with calculated Brunauer-Emmett-Teller (BET) surface area and total pore volume being 11.7 and 0.034 cm^3^·g^−1^, respectively.

### 2.2. Performance of MNP@MPTMS-VPA-Ti(IV) for Phosphoprotein Enrichment

To investigate the optimal loading conditions for intact phosphoprotein enrichment, the percentage of trifluoroacetic acid (TFA) and acetonitrile (CH_3_CN) in the loading buffer was optimized. Based on loading conditions reported in the literature [23,26] and preliminary experimental results, six different TFA percentages (0, 0.02%, 0.05%, 0.1%, 0.5%, and 1%) and CH_3_CN percentages (40%, 50%, 60%, 70%, 80%, and 90%) were selected. A mixed protein solution (20 µg·mL^−1^) containing BSA and *β*-CN (1:1 ratio) was loaded onto the MSPE adsorbent and eluted using the same eluent (12.5% NH_3_ aqua—50% CH_3_CN—37.5% H_2_O). As shown in Figures S7 and S8, at lower TFA percentages (0%, 0.02%), the material exhibited good enrichment capability for *β*-CN. However, peaks corresponding to *κ*-CN (19 kDa, M^+^), which has few phosphorylated sites, and non-phosphorylated protein BSA (66 kDa, M^+^; 33 kDa, M^2+^) also appeared in the eluate, indicating adsorption of these proteins by the material. This phenomenon is likely due to the nonspecific adsorptions of the nanomaterial to these proteins, including the electrostatic interaction between ionized carboxyl –COO^−^ of BSA and Ti^4+^ on the material. As the TFA percentage increased (0.05%, 0.1%), the appropriate acidity minimized the nonspecific adsorption. Consequently, the selectivity of the material for phosphoproteins improved while maintaining good enrichment efficiency. When the acid percentage further increased (0.5%, 1%), MNP@MPTMS–VPA–Ti(IV) still showed good selectivity for intact phosphoproteins, but the enrichment efficiency was inferior to that achieved at 0.1% TFA. This decrease may be attributed to the impact of high acid concentration on the stability of the material. For CH_3_CN, the enrichment efficiency improved when the percentage rose to 80% and showed a decrease when the percentage kept rising, as shown in Figures S9 and S10. Therefore, considering both selectivity and enrichment efficiency, a loading buffer with 80% CH_3_CN and 0.1% TFA was selected for further experiment.

**Figure 3:**
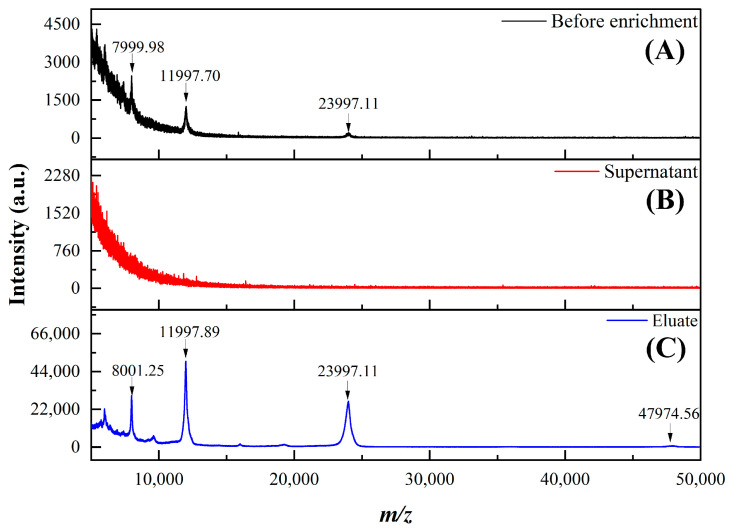
MALDI-TOF MS spectra of intact *β*-CN: before enrichment (**A**), supernatant (**B**), and eluate (**C**).

**Figure 4:**
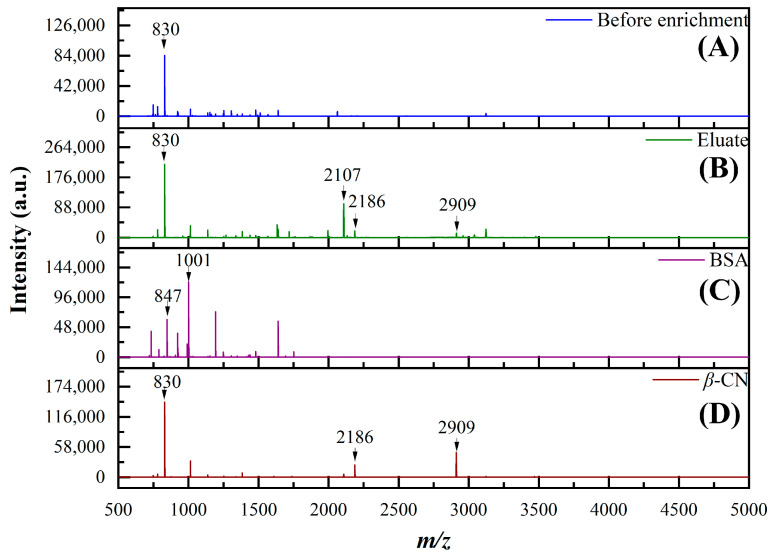
MALDI-TOF MS spectra of different tryptic digests: protein mixture before enrichment (20 µg·mL^−1^) (**A**), eluate (**B**), 100 µg·mL^−1^ BSA (**C**), and 100 µg·mL^−1^ *β*-CN (**D**).

Based on the optimized conditions established, the enrichment efficiency of MNP@MPTMS–VPA–Ti(IV) for intact phosphoproteins was evaluated using intact *β*-CN as a model protein, combined with MALDI-TOF MS analysis. Figure 3 displays the MALDI-TOF MS spectra of the solution before enrichment, the post-adsorption supernatant, and the eluate. The results show that the signal of the peak corresponding to *β*-CN (24 kDa) is weak before enrichment. No *β*-CN-related peaks are detectable in the supernatant after adsorption, indicating nearly complete adsorption of *β*-CN by the material. In the spectrum of the eluate, the peak intensity of *β*-CN is significantly enhanced. To further investigate the enrichment efficiency of the material for *β*-CN, the solutions before and after enrichment were subjected to enzymatic digestion (Figure 4). Using 100 µg·mL^−1^ digests of BSA and *β*-CN as references, the digests were also analyzed by MALDI-TOF MS. It was found that only one relatively distinct *β*-CN peak (*m*/*z* 830) appeared before enrichment. In contrast, the intensities of several *β*-CN peptide peaks in the eluate (*m*/*z* 830, 2186, 2909; specific sequences refer to Table S1) were markedly enhanced. Notably, no BSA peptide peaks were detected in the eluate. These results confirm the selectivity and considerable enrichment capability of the material for intact phosphoproteins.

To investigate the limit of detection (LOD) for intact phosphoprotein enrichment using MNP@MPTMS–VPA–Ti(IV), the material was added to *β*-CN solutions at four different concentrations (10, 5, 2, and 1 µg·mL^−1^). An identical amount of material (50 µL, 20 mg·mL^−1^) was used for phosphoprotein enrichment. As shown in Figures S11 and S12, the *β*-CN peak remained observable even at a concentration as low as 2 µg·mL^−1^ after enrichment and elution. This verifies that the material achieves highly sensitive enrichment of low-concentration intact phosphoproteins.

Given the potential interference from high-abundance non-phosphorylated proteins in real samples, the selectivity of MNP@MPTMS–VPA–Ti(IV) for intact phosphoprotein enrichment need to be evaluated. Therefore, the mixtures of BSA and *β*-CN solutions at different mass ratios (20:1, 50:1, 100:1, 200:1) were prepared. An identical amount of material (50 µL, 20 mg·mL^−1^) was added for phosphorylated protein enrichment, followed by analysis using MALDI-TOF MS (Figures S13 and S14). At a ratio of 20:1 (*w*/*w*), compared to the numerous BSA-related peaks (66 kDa) in the loading solution, the eluate primarily contained only *β*-CN-related peaks (24 kDa). As the ratio increased to 50:1 and 100:1, the BSA peaks before enrichment became more pronounced, even overshadowing the *β*-CN peak. However, *β*-CN peaks remained dominant in the eluate, although some BSA-related peaks (e.g., 13.5 kDa, M^5+^) appeared. When the ratio rose to 200:1, no distinct protein peaks were clearly observed in the eluate. The adsorption of phosphoproteins by the material was likely compromised by interference from the high concentration of non-phosphorylated protein. The material retains high selectivity for enriching intact phosphoproteins even at a *β*-CN to BSA mass ratio of 1:100, demonstrating good enrichment selectivity.

As a key factor in magnetic nanocomposites, the reusability and stability of MNP@MPTMS–VPA–Ti(IV) were evaluated by washing with the loading buffer several times before every enrichment of *β*-CN. As shown in Figure S15, the MALDI-TOF spectra of the eluate maintained similar peaks and intensity after three times of enrichment by the same batch of MNP@MPTMS–VPA–Ti(IV). This indicates that the nanomaterial showed good reusability and stability for the enrichment of intact phosphoproteins.

### 2.3. Enrichment of Phosphoproteins in Real Samples by MNP@MPTMS-VPA-Ti(IV)

**Figure 5:**
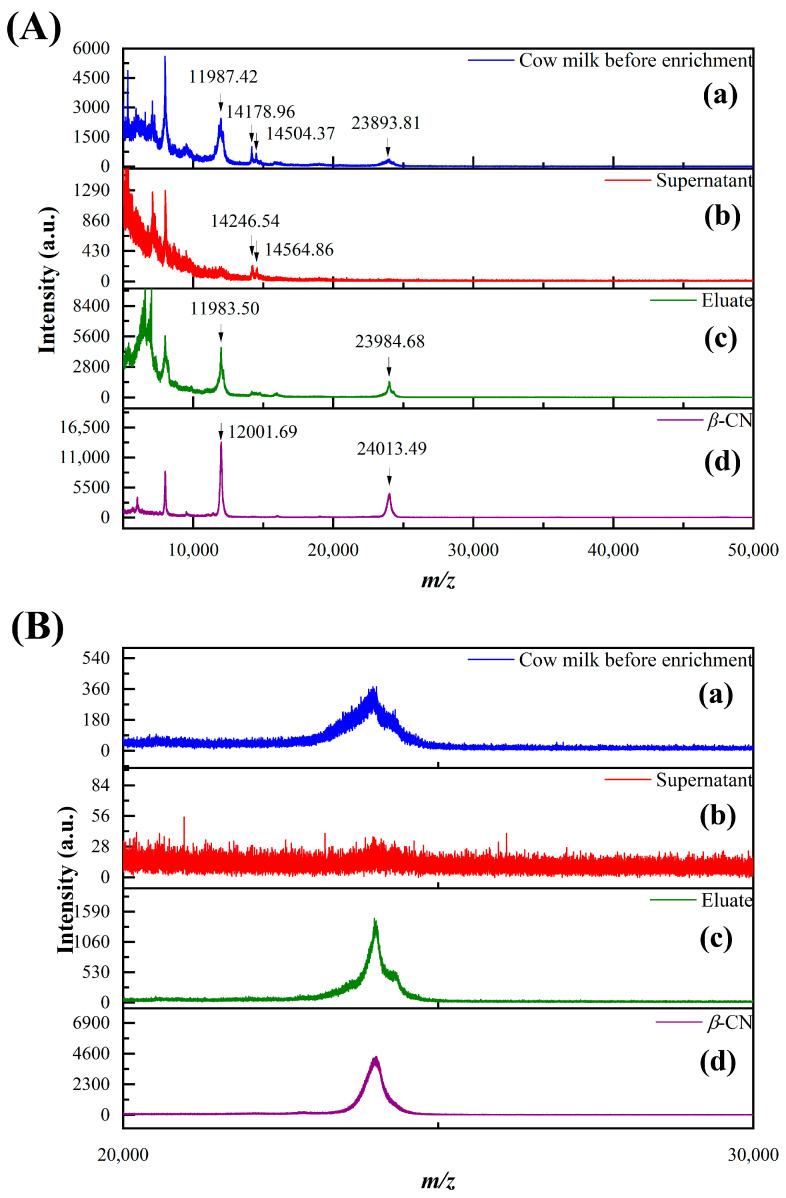
MALDI-TOF MS spectra (**A**) and enlarged spectra at 20–30 kDa (**B**) of milk samples: before enrichment (**a**), supernatant (**b**), eluate (**c**), and 100 µg·mL^−1^ *β*-CN (**d**).

The main proteins present in milk include caseins (CN), whey proteins (*α*-lactalbumin, *β*-lactoglobulin), bovine serum albumin (BSA), etc. [48]. Among these, caseins are typical phosphoproteins with several proteins identified as allergenic from casein fractions [41], while whey proteins and bovine serum albumin lack phosphorylation modifications. Although the content of caseins is relatively high in milk [49], direct mass spectrometric detection of intact caseins in milk yields weak signals due to interference from matrix components such as carbohydrates and lipids, as well as other high-abundance proteins (whey proteins). Therefore, non-fat milk was selected as a real sample to investigate the enrichment of intact casein using MNP@MPTMS–VPA–Ti(IV), combined with MALDI-TOF MS analysis. This preliminarily verified the capability of the material to enrich intact phosphoproteins in real samples. As seen in Figure 5a, the milk sample before enrichment exhibited peaks corresponding to caseins (24 kDa, M^+^; 12 kDa, M^2+^; 8 kDa, M^3+^) and *α*-lactalbumin (14 kDa, M^+^), but the signal of the 24 kDa casein peak was weak. The supernatant after adsorption primarily contained peaks of *α*-lactalbumin (Figure 5b), further confirming the selectivity of the material for phosphoproteins. In the eluate (Figure 5c), the *α*-lactalbumin peak was weakened, while the peak intensity of caseins was markedly enhanced (approximately 4.5 times stronger than before enrichment). Furthermore, compared to the MS spectra of pre-enriched milk (Figure 5a) and *β*-CN (Figure 5d), the peaks corresponding to three different caseins could be resolved (23.5, 24, and 24.5 kDa corresponding to *α*_S1_-CN, *β*-CN, and *α*_S2_-CN, respectively).

**Figure 6:**
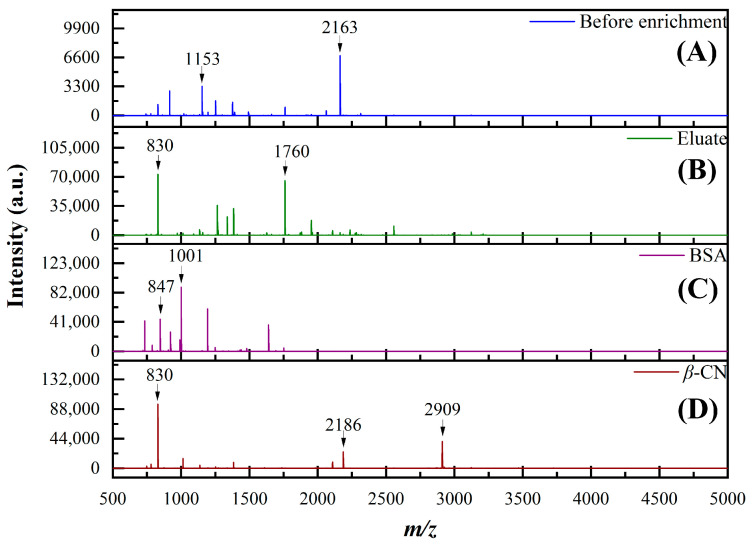
MALDI-TOF MS spectra of different tryptic digests: milk sample before enrichment (**A**), eluate (**B**), 100 µg·mL^−1^ BSA (**C**), and 100 µg·mL^−1^ *β*-CN (**D**).

Consistent with the previous evaluation of model protein enrichment efficiency, considering the low resolution of mass spectrometry for detecting large molecular weight proteins, the solutions before and after enrichment were further subjected to enzymatic digestion. Combined with MALDI-TOF MS analysis, the enrichment efficiency of the material for caseins from milk was further verified at the peptide level (Figure 6). Table S2 displays the position, sequence and theoretical mass of related peptides. Likely due to low concentration of protein before enrichment, peptides such as *m*/*z* 1153 and 2163 appeared in the peptide mass spectrum, which were identified as the digest of trypsin itself according to database search (corresponding to sequences SSGTSYPDVLK and LGEDNINVVEGNEQFISASK, respectively). In contrast, the digest of the eluate exhibited high-intensity peaks at *m*/*z* 830 (AVPYPQR, originating from *β*-CN) and *m*/*z* 1760 (HQGLPQEVLNENLLR, originating from *α*_S1_-CN). In contrast, no peptide peaks corresponding to BSA or whey proteins were observed. This further demonstrates the enrichment efficiency and selectivity of MNP@MPTMS–VPA–Ti(IV) for intact phosphorylated proteins in real samples, confirming the applicability of the material.

**Figure 7:**
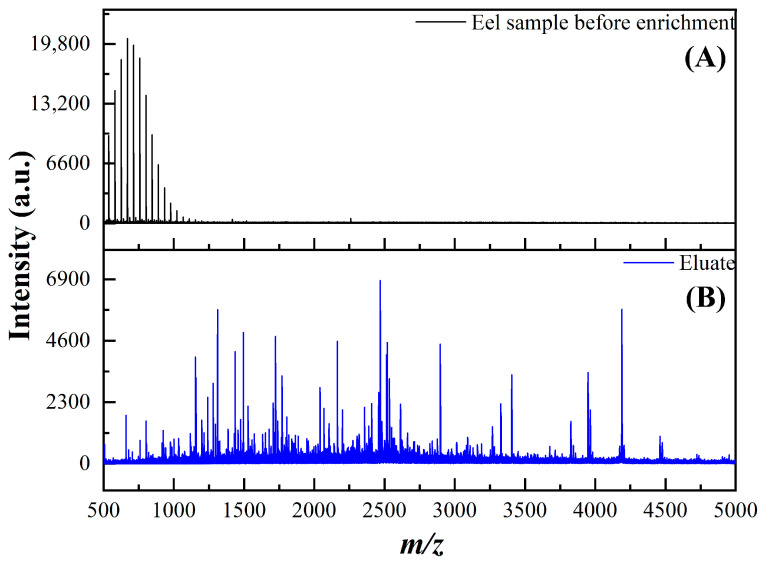
MALDI-TOF MS spectra of tryptic digests of proteins from the eel mucus sample: before (**A**) and after (**B**) enrichment.

Nowadays, food-borne pathogens pose a threat to consumer health due to the toxicity of their metabolites and themselves. The presence and status of pathogens may cause different expression of proteins in the organisms they parasitize [50,51,52]. As exemplified by eel, infection with anguillid herpesvirus may cause the upregulation of some phosphoproteins, such as DSP (A0A8M9PQ61), STAT1 (O93598), CAV-1 (Q6YLH9), and CTNN*β*1 (F1QGH7) [52]. However, the detection of intact proteins may be affected by a complex organism. Therefore, we applied the prepared nanocomposite for enrichment in eel mucus to verify its applicability in complex samples. The pretreatment process of the skin mucus of the eel is given in the Supplementary Materials according to [51]. Considering the low resolution of MS for intact proteins in eel, the digests of the proteins in the mucus sample before and after enrichment by MNP@MPTMS–VPA–Ti(IV) were compared and analyzed, as shown in Figure 7. Before enrichment, the MS spectrum of the digests of un-enriched proteins in mucus shows a series of peaks with an interval of *m*/*z* 43, which might be attributed to polyethylene glycol introduced into the system during the extraction of proteins. For eluate, no such peaks were observed. The peptides in the spectrum of the digests of enriched proteins from the mucus sample were searched in the MASCOT database and correspond to ankyrin repeat and fibronectin type-III domain-containing protein 1 (ANKF1_DANRE, A0A8M9QN10), showing a coverage of 43%. Related peptides and sequences in the eluate are listed in Table S3, with corresponding phosphorylated sites. The protein is expressed by the ankfn1 gene and is required for vestibular-related functions, according to the Uniprot database. The results indicate successful enrichment of intact phosphoprotein in complex eel samples with the prepared material MNP@MPTMS–VPA–Ti(IV), showing its potential for the enrichment of pathogen-related phosphoproteins.

## 3. Materials and Methods

### 3.1. Materials and Reagents

Sodium acetate anhydrous (CH_3_COONa), titanium(IV) sulfate (Ti(SO_4_)_2_), and ethanol (CH_3_CH_2_OH) were purchased from Sinopharm Chemical Reagent Co., Ltd. (Shanghai, China). Ethylene glycol (EG) was obtained from Shanghai No. 4 Chemical Reagent Co., Ltd. (Shanghai, China). 2,2′-Azobis(2-methylpropionitrile) (AIBN), ammonia solution (NH_3_ aqua), ammonium bicarbonate (NH_4_HCO_3_), and acetonitrile (CH_3_CN) were purchased from Macklin Biochemical Co., Ltd. (Shanghai, China). Formic acid (HCOOH) and 2,5-dihydroxybenzoic acid (DHB) were obtained from TCI Chemicals (Shanghai, China). Vinylphosphonic acid (VPA), trifluoroacetic acid (TFA), and phosphoric acid (H_3_PO_4_) were purchased from Aladdin Biochemical Technology Co., Ltd. (Shanghai, China). 3-Mercaptopropyltrimethoxysilane (MPTMS) was purchased from Innochem Science & Technology Co., Ltd. (Beijing, China). Iron(III) chloride hexahydrate (FeCl_3_·6H_2_O) was purchased from CNS Technology Co., Ltd. (Tianjin, China). *α*-Cyano-4-hydroxycinnamic acid (CHCA), *β*-casein from bovine milk (*β*-CN), bovine serum albumin (BSA), and trypsin were purchased from Sigma-Aldrich (St. Louis, MO, USA). Ultrapure water was obtained from the Milli-Q Ultrapure Water Preparation System from Millipore (Burlington, MA, USA). Non-fat milk and eel were purchased from the local retailers. Among the above reagents, TFA and CH_3_CN were of HPLC grade. All other reagents were of analytical grade and were used without further purification.

### 3.2. Synthesis of MNP@MPTMS

Fe_3_O_4_ nanoparticles were first fabricated via the solvothermal reaction method [53]. Sodium acetate (3.6 g) was dispersed in 40 mL of ethylene glycol. The mixture was ultrasonicated and vortexed to form a clear, transparent solution. Then, FeCl_3_·6H_2_O (1.35 g, 5 mmol) was added to the system and mixed uniformly by sonication to form a yellow-brown solution. The solution was then transferred to a 50 mL Teflon-lined autoclave, sealed in a stainless-steel reaction vessel and reacted at 200 °C for 8 h. After cooling to room temperature, the magnetic particles were separated with an external magnetic field and washed sequentially with ultrapure water and ethanol three times each. The washed nanoparticles were dried under vacuum at 60 °C for 12 h. Then, the synthesized Fe_3_O_4_ (200 mg) was weighed and dispersed in 150 mL of an ethanol-aqueous solution with 8 mL NH_3_ aqua. Under ultrasonic treatment and vigorous stirring, 1 mL MPTMS was divided into two batches and added drop by drop to the reaction every 5 h. The resulting MNP@MPTMS nanocomposites were separated magnetically and washed three times with water and ethanol.

### 3.3. Synthesis of MNP@MPTMS-VPA-Ti(IV)

MNP@MPTMS (100 mg) particles were dispersed in 50 mL ethanol solution, followed by the addition of 40 mg AIBN and ultrasonic homogenization. The mixture was transferred to a 250 mL three-necked flask, supplemented with 50 mL ethanol and 500 µL vinylphosphonic acid (VPA). The reaction proceeded at 60 °C for 24 h under N_2_ atmosphere. The obtained MNP@MPTMS-VPA was dispersed in 20 mL 100 mM Ti(SO_4_)_2_ solution (containing 0.1% formic acid, *v*/*v*) and reacted under magnetic stirring for 8 h. The resulting MNP@MPTMS-VPA-Ti(IV) nanocomposites were separated via external magnetic field, then washed three times with formic acid (0.1%, *v*/*v*).

### 3.4. Selective Enrichment of Intact Phosphoproteins with MNP@MPTMS-VPA-Ti(IV) from Different Samples

The synthesized MNP@MPTMS-VPA-Ti(IV) nanoparticles were dispersed in a loading buffer (80% CH_3_CN—0.1% TFA—19.9% H_2_O) to form a 20 mg·mL^−1^ suspension. The material dispersion (50 µL, 1 mg) was added to the 200 µL sample solution and vortexed for 4 h to ensure sufficient interaction and adsorption between proteins and the functionalized material. The mixture was then separated using an external magnetic field and washed three times with 200 µL of loading buffer to remove nonspecifically-adsorbed proteins. Subsequently, the elution buffer (10 µL, 12.5% NH_3_ aqua—50% CH_3_CN—37.5% H_2_O) was added and vortexed for 20 min. The protein eluate was collected via external magnetic field separation and subjected to subsequent MALDI-TOF MS analysis.

### 3.5. MALDI-TOF MS Analysis

After enrichment with MNP@MPTMS-VPA-Ti(IV), the eluate was dropped on a MTP 384 target plate, polished steel BC, and then 1.0 μL of (*α*-cyano-4-hydroxycinnamic acid, CHCA) aqueous solution (10 mg·mL^−1^, 50% CH_3_CN—1%H_3_PO_4_) was added as a matrix. MS analysis was carried out on an ultrafleXtreme MALDI TOF/TOF mass spectrometer system (Bruker Daltonics, Bremen, Germany) with a Nd-YAG laser emitting at 355 nm in linear positive ion mode and an acceleration voltage of 20 kV.

## 4. Conclusions

In summary, a Ti-IMAC nanocomposite aiming at phosphoproteins, MNP@MPTMS–VPA–Ti(IV), was developed in this work. Using Fe_3_O_4_ synthesized via the solvothermal method as the base substrate, it was coated with a mercaptosilane. Subsequently, phosphonic acid groups were grafted onto the material via a thiol-ene click reaction to chelate and immobilize Ti^4+^ ions. The reaction conditions were mild, aligning with the principles of green chemistry. The nanomaterial was then employed as an MSPE adsorbent to separate and enrich intact phosphoproteins based on the top-down strategy. Combined with MALDI-TOF MS analysis, the method was optimized and evaluated. The nanocomposite showed a low limit of detection (2 µg·mL^−1^), strong anti-interference (BSA:*β*-CN = 100:1), and good reusability. It was successfully applied for the enrichment of intact phosphoproteins even in complex samples like milk and eel, which indicates the material can serve as a promising candidate for the pretreatment of biological samples in phosphoproteomic analysis.

## Data Availability

Data are contained within the article and Supplementary Materials.

## Supplementary Materials

The following supporting information can be downloaded at: https://www.mdpi.com/article/10.3390/molecules31030396/s1. Characterization; Preparation of non-fat milk samples; Preparation of eel mucus samples; Enzymatic digestion; Figure S1: Size distribution diagram of MNP@MPTMS–VPA–Ti(IV); Figure S2: PXRD patterns of MNP, MNP@MPTMS, MNP@MPTMS–VPA, MNP@MPTMS–VPA–Ti(IV); Figure S3: XPS segmented spectra of S 2p and P 2p; Figure S4: EDS spectrum of MNP@MPTMS–VPA–Ti(IV); Figure S5: Magnetization curves of MNP, MNP@MPTMS–VPA and MNP@MPTMS–VPA–Ti(IV); Figure S6: BET curves of MNP@MPTMS–VPA–Ti(IV); Figure S7: MALDI-TOF MS spectra of 20 µg·mL^−1^ *β*-CN and BSA protein mixture (1:1) enriched by MNP@MPTMS–VPA–Ti(IV) with different loading buffer; Figure S8: Intensity comparison of the peaks of *β*-CN enriched by MNP@MPTMS–VPA–Ti(IV) with different loading buffer; Figure S9: MALDI-TOF MS spectra of 20 µg·mL^−1^ *β*-CN and BSA protein mixture (1:1) enriched by MNP@MPTMS–VPA–Ti(IV) with different loading buffer; Figure S10: Intensity comparison of the peaks of *β*-CN enriched by MNP@MPTMS–VPA–Ti(IV) with different loading buffers; Figure S11: MALDI-TOF MS spectra of intact *β*-CN at different mass concentrations enriched with MNP@MPTMS–VPA–Ti(IV); Figure S12: Intensity comparison of intact *β*-CN at different mass concentrations of 10 µg·mL^−1^ (A), 5 µg·mL^−1^ (B), 2 µg·mL^−1^ (C), and 1 µg·mL^−1^ (D) enriched with MNP@MPTMS–VPA–Ti(IV); Figure S13: MALDI-TOF MS spectra of *β*-CN and BSA protein mixtures at different mass ratios enriched with MNP@MPTMS–VPA–Ti(IV); Figure S14: Intensity comparison of *β*-CN enriched by MNP@MPTMS–VPA–Ti(IV) from protein mixtures (*β*-CN:BSA) at different mass ratios of 1:20 (A), 1:50 (B), 1:100 (C), and 1:200 (D); Figure S15: Reusability test of MNP@MPTMS–VPA–Ti(IV) in enrichment of intact *β*-CN from 20 µg·mL^−1^ *β*-CN and BSA protein mixture (1:1); Table S1: Position, sequence and theoretical mass of corresponding peptides in tryptic digests of *β*-CN and BSA; Table S2: Position, sequence and theoretical mass of corresponding peptides in tryptic digests of caseins from bovine milk and BSA; Table S3: Position, sequence and theoretical mass of corresponding peptides in tryptic digests of eel sample eluate.
