# Supplementary material for: Preparation of a Magnetic Ti-IMAC Material Based on Thiol-Ene Click Reaction and the Application in Intact Phosphoprotein Enrichment
Source: Molecules. 2026 Jan 23;31(3):396. doi: 10.3390/molecules31030396 (PMC12898470; doi:10.3390/molecules31030396)
Supplement: Supplementary file 1 [file molecules-31-00396-s001.zip › molecules-4064940-supplementary.pdf]

Supplementary Material

Preparation of a Magnetic Ti-IMAC Material Based on Thiol-Ene Click Reaction and the Application in Intact Phosphoprotein Enrichment

Yan Lu [1], Sen Zhang [1], Hong-Yan Ge [1], Han-Yue Yang [2,*], Feng Zhang [1], Yi-Fan Pan [1] and Hong-Zhen Lian [1,*]

[1] State Key Laboratory of Analytical Chemistry for Life Science, Jiangsu Key Laboratory of Clean Energy Catalysis and Intelligent Green Chemical Engineering, School of Chemistry & Chemical Engineering and Center of Materials Analysis, Nanjing University, Nanjing 210023, China; ly181850106@163.com (Y.L.)

[2] Jiangsu Deyuan Pharmaceutical Co., Ltd., 21 Jinqiao Road, Lianyungang 222002, China

* Correspondence: hzlian@nju.edu.cn (H.-Z.L.); yanghanyue01@163.com (H.-Y.Y.)

Supplementary experiment section

Characterization. The scanning electron microscopy (SEM) images and SEM-energy dispersive spectrometer (SEM-EDS) results of the materials were taken on a JSM-7800 F electron microscope (JEOL, Tokyo, Japan). The transmission electron microscopy (TEM) images of the microspheres were obtained using a Jem-2100F transmission electron microscope (JEOL, Tokyo, Japan). ATR-FTIR spectra of materials were recorded on a Nicolet 6700 Fourier Transform Infrared spectrometer (Thermo Nicolet Corporation, WI, USA). Raman spectra were recorded on a Renishaw inVia-Reflex Raman spectrometer (Renishaw, London, UK). Powder X-ray diffraction (PXRD) patterns were evaluated on D8 Advance X-ray diffractometer (Bruker, Germany) with Cu Kα radiation. X-ray photoelectron spectroscopy (XPS) analysis was carried out on a Nexsa Probe photoelectron spectrometer (Thermo Fisher, MA, USA). ζ-potential measurements were carried out using Nano-Z Zeta Potential Analyzer (Malvern, UK). The magnetic properties (VSM) of materials were measured by a Lake Shore 8604 vibrating sample magnetometer (Lake Shore, OH, USA) at room temperature. N_2 adsorption-desorption isotherms were carried out on a Micromeritics ASAP 2020 BET surface analyzer system (Micromeritics, Shanghai, China). The surface area was calculated by using the Brunauer-Emmett-Teller (BET) equation, and pore size distribution was obtained by using the Barrett-Joyner-Halenda (BJH) model.

Preparation of non-fat milk samples. Take 30 μL of milk sample and add 970 μL NH_4HCO_3 solution (50 mM). The solution was centrifuged at 14,000 rpm and 4°C for 10 minutes and the supernatant was collected (approx. 1 mg/mL). Transfer 50 μL of supernatant, add 950 μL of loading buffer (80% ACN - 0.1% TFA - 19.9% H_2O), followed by 50 μL of material dispersion (20 mg/mL, 1 mg). After vortex for 4 hours, magnetic separation was performed and the supernatant was retained.

Preparation of eel mucus samples. The skin mucus of eel samples were collected and ground with liquid nitrogen. Then the organism was dispersed in the lysis buffer (8M urea, 1% Protease Inhibitor Cocktail) and ultrasonicated for 20 min. The solution was centrifuged at 14000 rpm and 4°C for 10 minutes and the supernatant was collected. In order to allow sufficient contact and interaction between the material and the samples, add 900 μL of loading buffer (80% ACN - 0.1% TFA - 19.9% H_2O) and 50 μL of material dispersion (20 mg/mL, 1 mg) to 100 μL supernatant obtained.

Enzymatic digestion. The eluate (10 μL) was evaporated to dryness in an evaporative concentrator at 25°C and reconstituted with 10 μL of NH_4HCO_3 solution. Then, trypsin(0.04 μg/μL, 5 μL) was added directly to prepared protein solution, followed by enzymatic digestion in a water bath at 37°C for 16 h. The digest (1.0 μL) was dropped on a MTP 384 target plate polished steel BC, and then 1.0 μL of 2,5-dihydroxybenzoic acid (DHB) aqueous solution (20 $mg \cdot mL^{-1}$, 50% ACN - 1% H_3PO_4) was added as a matrix. MS analysis was carried on a ultrafleXtreme MALDI

TOF/TOF mass spectrometer system (Bruker Daltonics, Germany) with Nd-YAG laser emitting at 355 nm in reflect positive ion mode and an acceleration voltage of 20 kV.

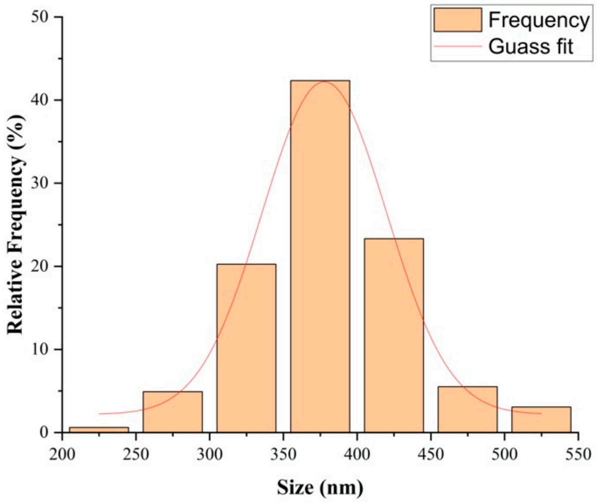

Figure S1. Size distribution diagram of MNP@MPTMS–VPA–Ti(IV).

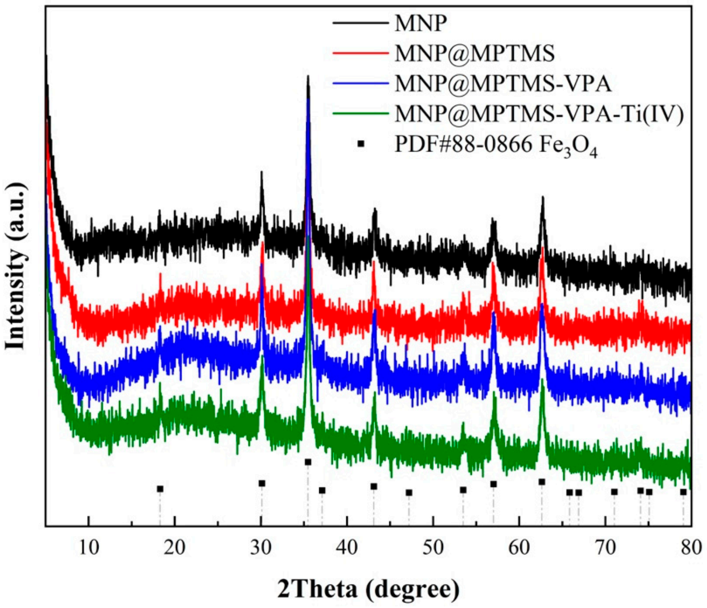

Figure S2. PXRD patterns of MNP, MNP@MPTMS, MNP@MPTMS–VPA, MNP@MPTMS–VPA–Ti(IV).

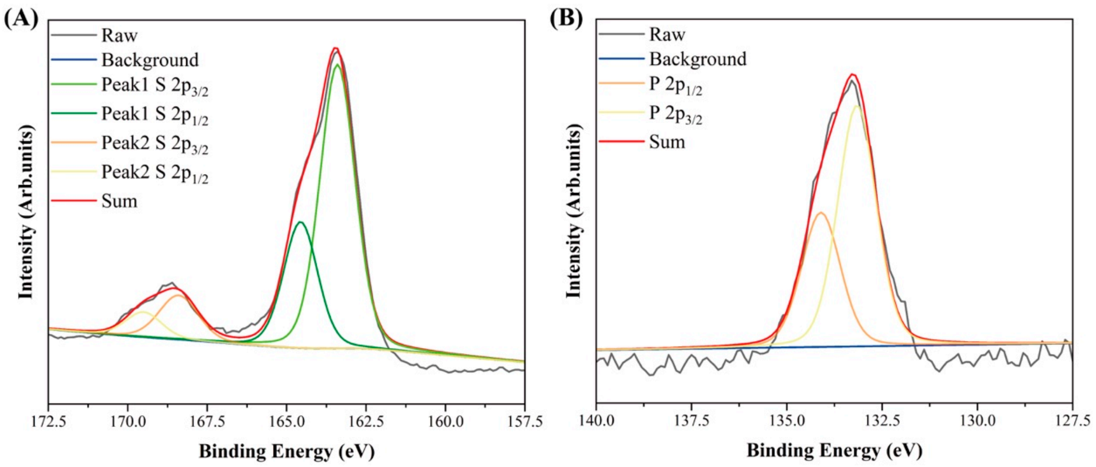

Figure S3. XPS segmented spectra of S 2p (A) and P 2p (B).

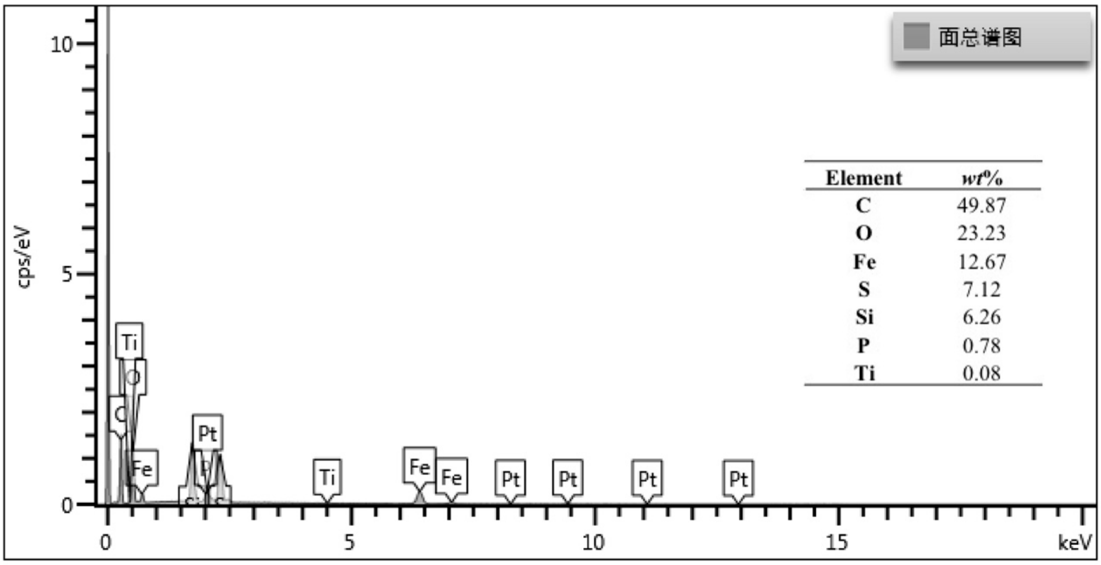

Figure S4. EDS spectrum of MNP@MPTMS–VPA–Ti(IV).

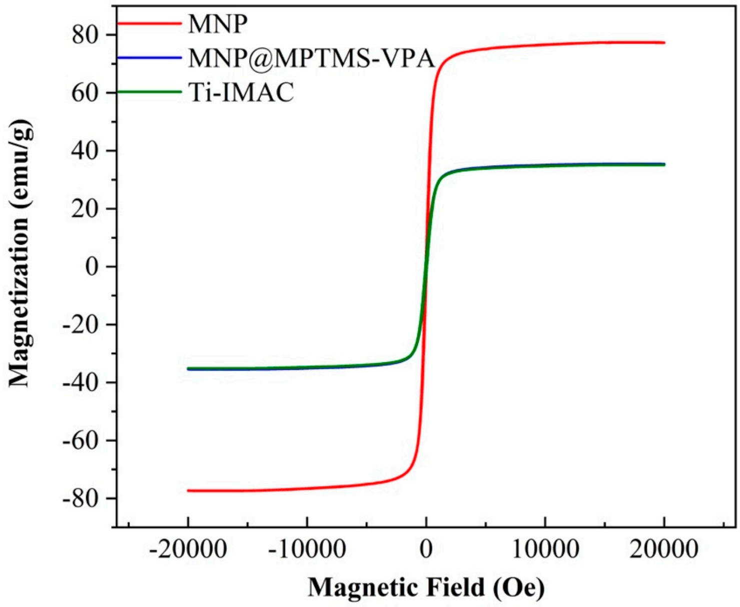

Figure S5. Magnetization curves of MNP, MNP@MPTMS–VPA and MNP@MPTMS–VPA–Ti(IV).

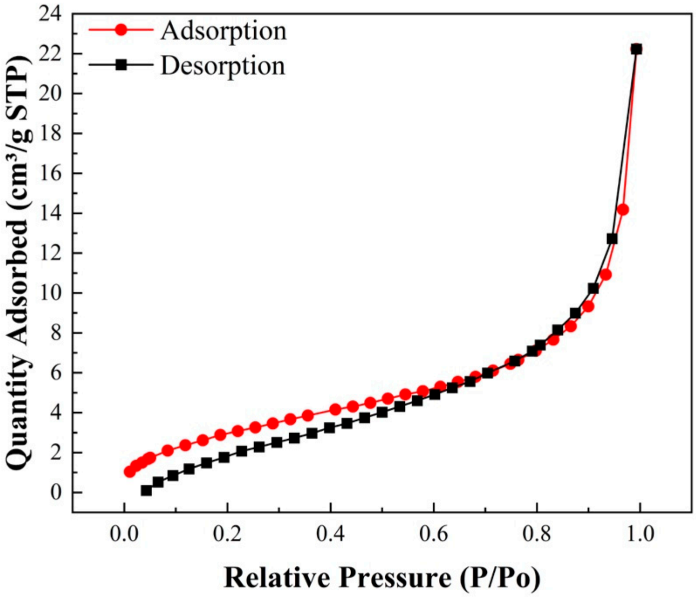

Figure S6. BET curves of MNP@MPTMS–VPA–Ti(IV).

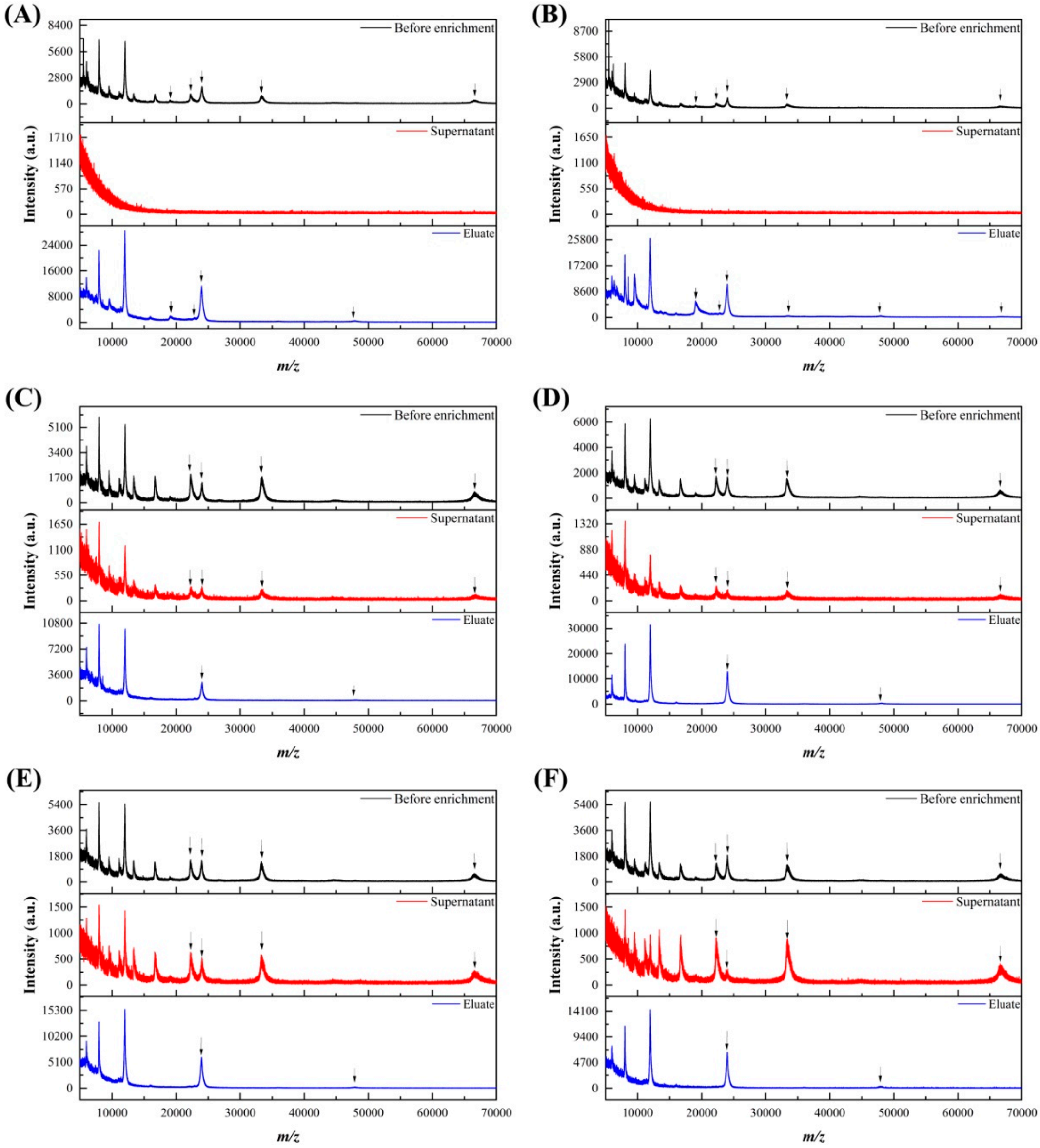

Figure S7. MALDI-TOF MS spectra of 20 μg·mL^{-1} β-CN and BSA protein mixture (1:1) enriched by MNP@MPTMS–VPA–Ti(IV) with different loading buffer: (A) 80% ACN + 0% TFA + 20% H_2O, (B) 80% ACN + 0.02% TFA + 19.98% H_2O, (C) 80% ACN + 0.05% TFA + 19.95% H_2O, (D) 80% ACN + 0.1% TFA + 19.9% H_2O, (E) 80% ACN + 0.5% TFA + 19.5% H_2O, (F) 80% ACN + 1% TFA + 19% H_2O.

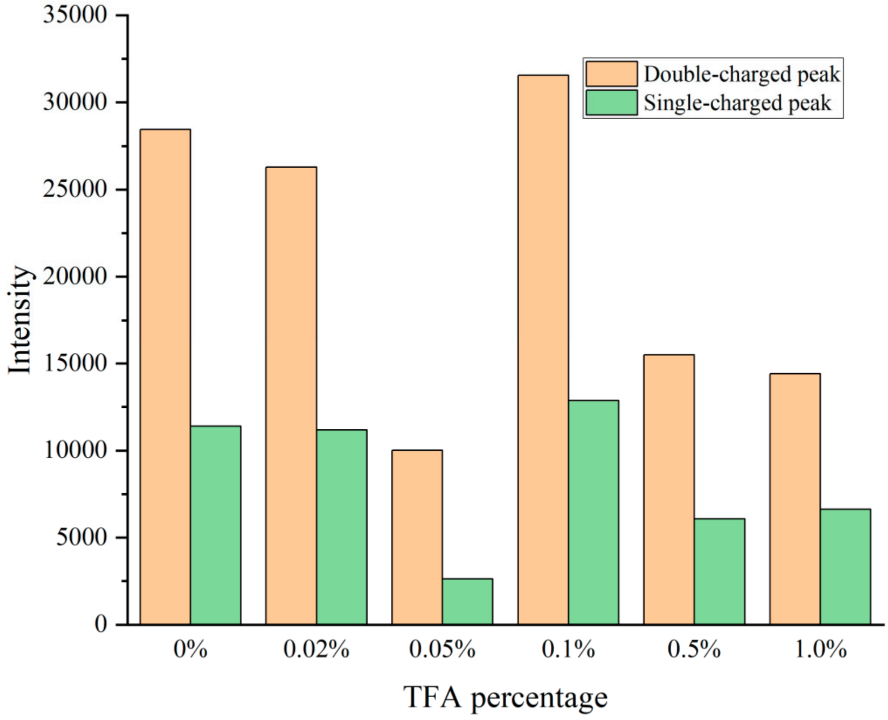

Figure S8. Intensity comparison of the peaks of *β*-CN enriched by MNP@MPTMS–VPA–Ti(IV) with different loading buffer: (A) 80% ACN + 0% TFA + 20% H_2O, (B) 80% ACN + 0.02% TFA + 19.98% H_2O, (C) 80% ACN + 0.05% TFA + 19.95% H_2O, (D) 80% ACN + 0.1% TFA + 19.9% H_2O, (E) 80% ACN + 0.5% TFA + 19.5% H_2O, (F) 80% ACN + 1% TFA + 19% H_2O.

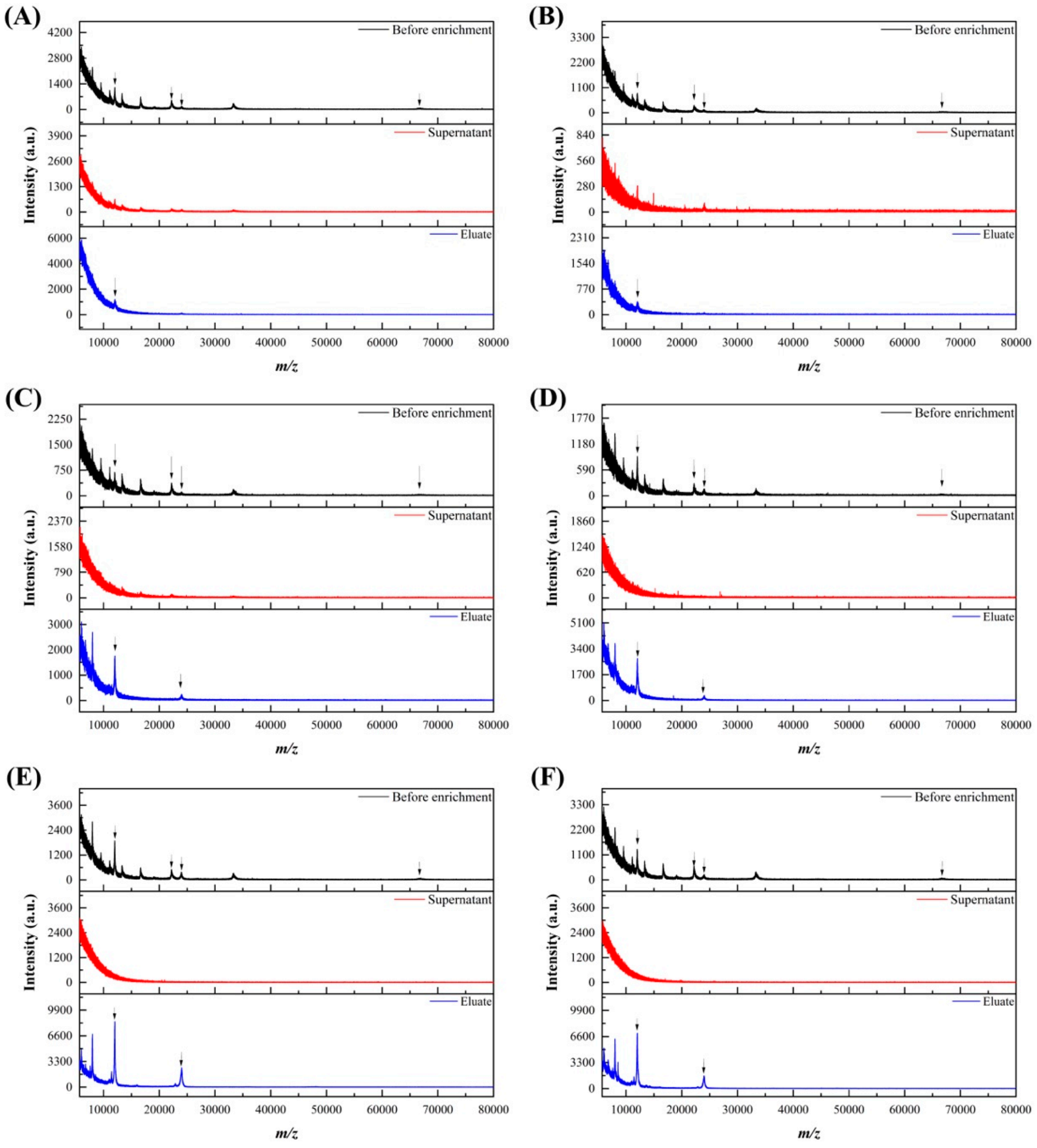

Figure S9. MALDI-TOF MS spectra of 20 μg·mL^{-1} *β*-CN and BSA protein mixture (1:1) enriched by MNP@MPTMS–VPA–Ti(IV) with different loading buffer: (A) 40% ACN + 0.1% TFA + 59.9% H_2O, (B) 50% ACN + 0.1% TFA + 49.9% H_2O, (C) 60% ACN + 0.1% TFA + 39.9% H_2O, (D) 70% ACN + 0.1% TFA + 29.9% H_2O, (E) 80% ACN + 0.1% TFA + 19.9% H_2O, (F) 90% ACN + 0.1% TFA + 9.9% H_2O.

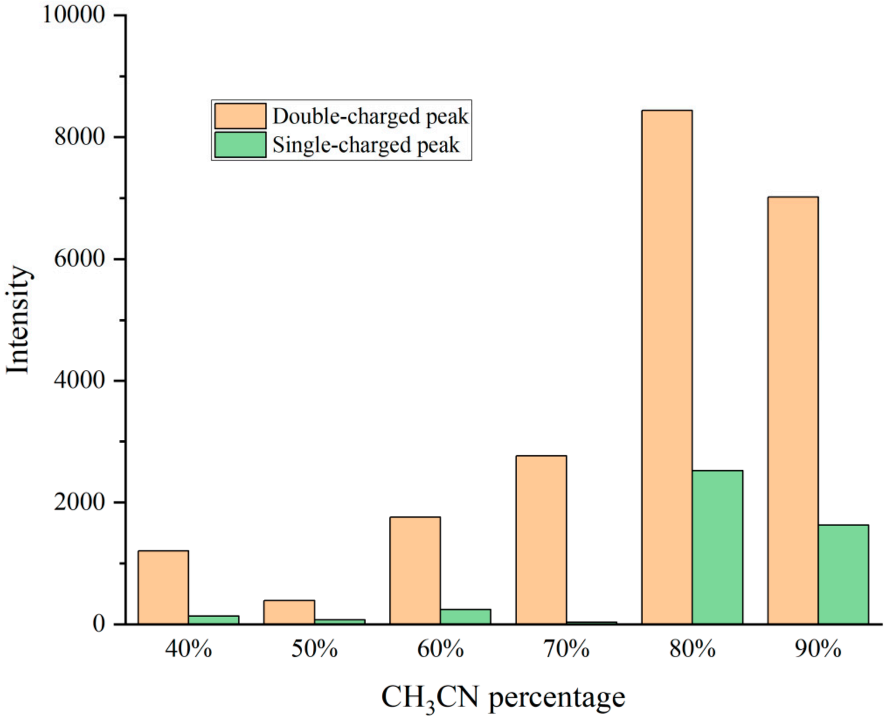

Figure S10. Intensity comparison of the peaks of *β*-CN enriched by MNP@MPTMS–VPA–Ti(IV) with different loading buffer: (A) 40% ACN + 0.1% TFA + 59.9% H_2O, (B) 50% ACN + 0.1% TFA + 49.9% H_2O, (C) 60% ACN + 0.1% TFA + 39.9% H_2O, (D) 70% ACN + 0.1% TFA + 29.9% H_2O, (E) 80% ACN + 0.1% TFA + 19.9% H_2O, (F) 90% ACN + 0.1% TFA + 9.9% H_2O.

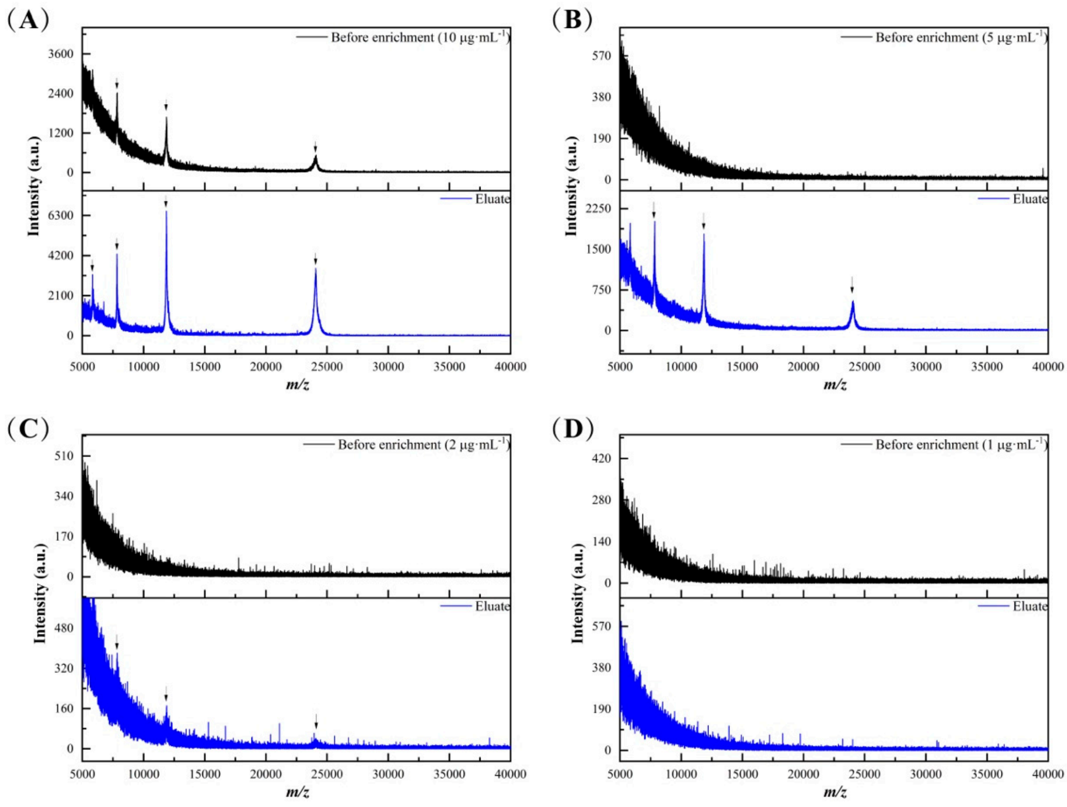

Figure S11. MALDI-TOF MS spectra of intact *β*-CN at different mass concentrations of 10 μg·mL^{-1} (A), 5 μg·mL^{-1} (B), 2 μg·mL^{-1} (C), 1 μg·mL^{-1} (D) enriched with MNP@MPTMS–VPA–Ti(IV).

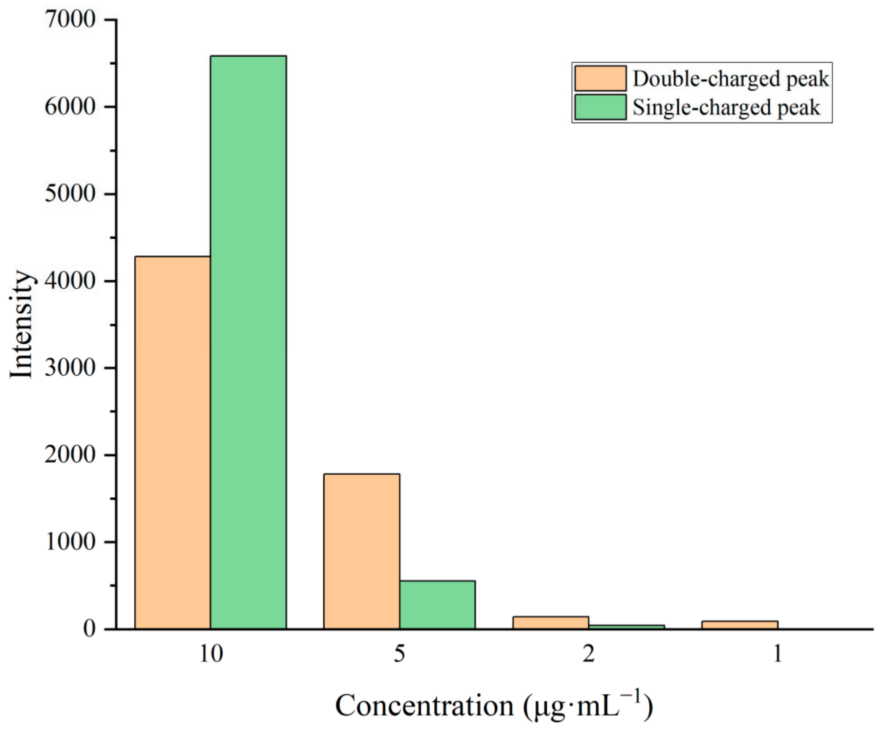

Figure S12. Intensity comparison of intact *β*-CN at different mass concentrations of 10 μg·mL^{-1} (A), 5 μg·mL^{-1} (B), 2 μg·mL^{-1} (C), 1 μg·mL^{-1} (D) enriched with MNP@MPTMS–VPA–Ti(IV).

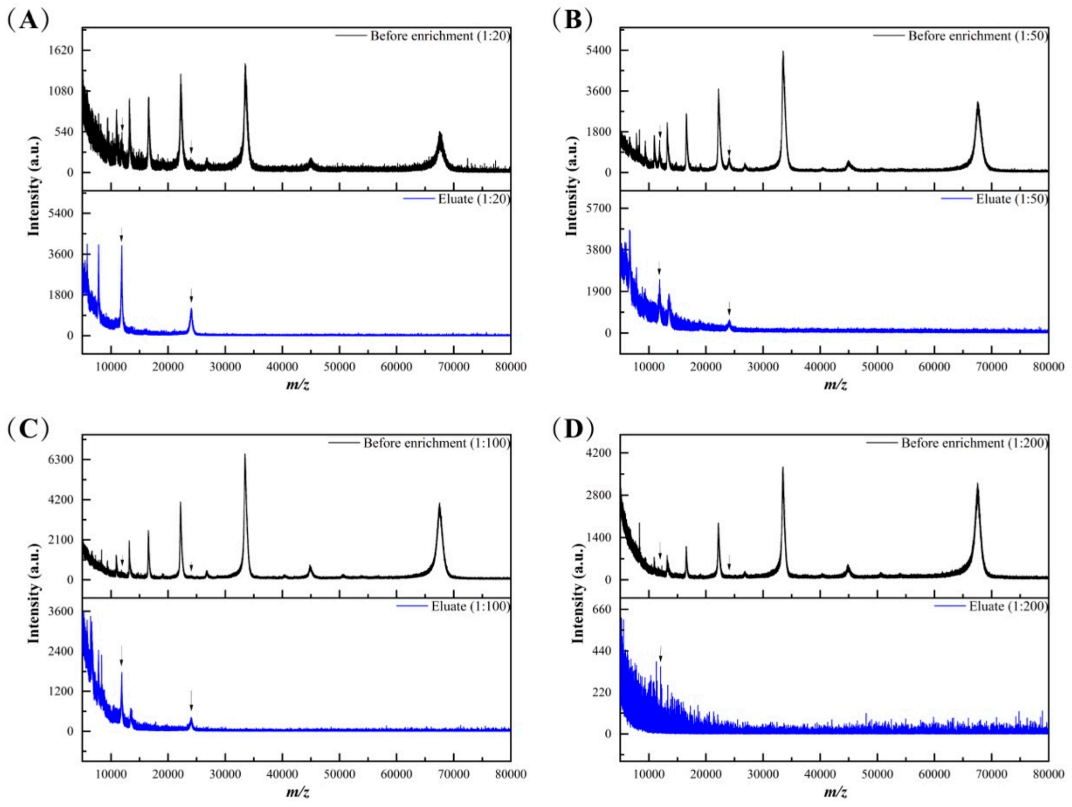

Figure S13. MALDI-TOF MS spectra of β-CN and BSA protein mixtures at different mass ratios of 1:20 (A), 1:50 (B), 1:100 (C), 1:200 (D) enriched with MNP@MPTMS–VPA–Ti(IV).

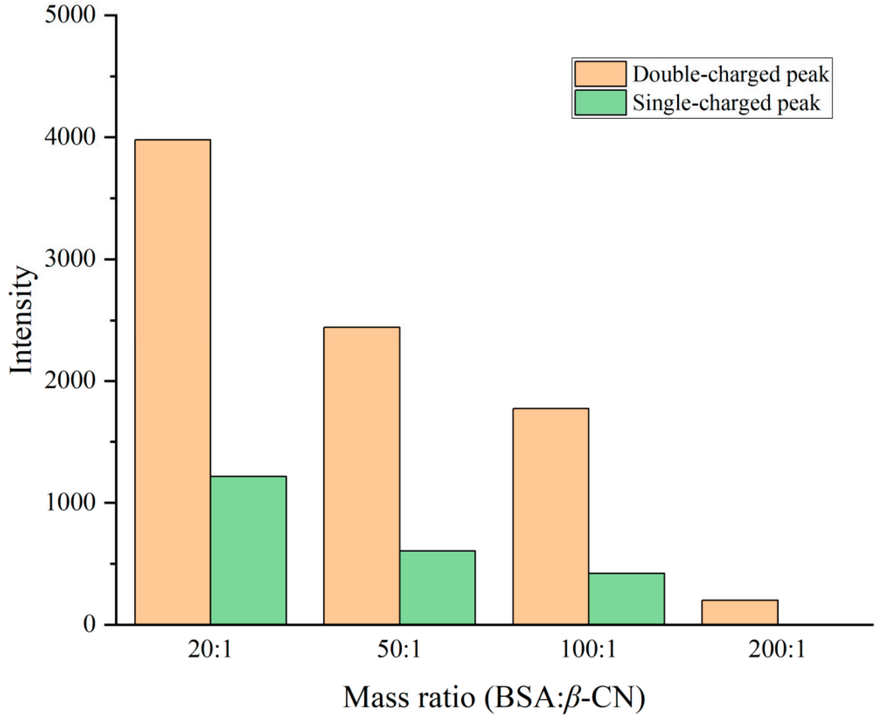

Figure S14. Intensity comparison of *β*-CN enriched by MNP@MPTMS–VPA–Ti(IV) from protein mixtures (*β*-CN:BSA) at different mass ratios of 1:20 (A), 1:50 (B), 1:100 (C), 1:200 (D).

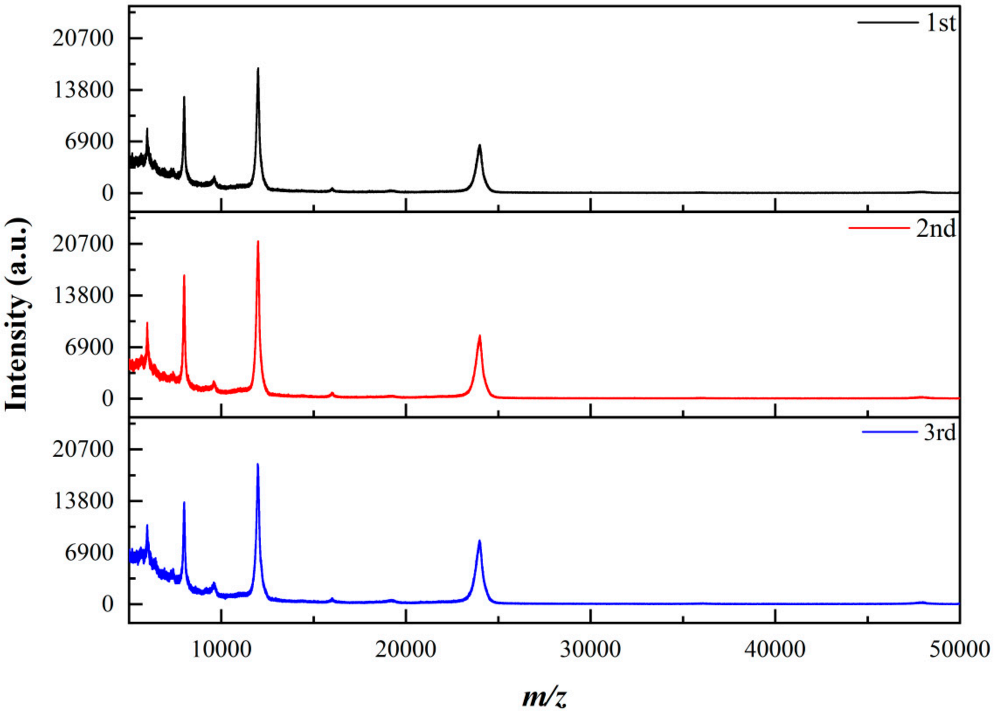

Figure S15. Reusability test of MNP@MPTMS–VPA–Ti(IV) in enrichment of intact β-CN from 20 μg·mL^{-1} β-CN and BSA protein mixture (1:1). From top to down: first, second, and third time.

Table S1. Position, sequence and theoretical mass of corresponding peptides in tryptic digests of *β*-CN and BSA.

Mass	Protein	Position	Sequence
847.5036	BSA	242-248	LSQKFPK
1001.5890	BSA	233-241	ALKAWSVAR
830.4519	*β*-CN	192-198	AVPYPQR
2186.1678	*β*-CN	199-217	DMPIQAFLLYQEPVLGPVR
2909.5997	*β*-CN	199-224	DMPIQAFLLYQEPVLGPVRGPFPIIV

Table S2. Position, sequence and theoretical mass of corresponding peptides in tryptic digests of caseins from bovine milk and BSA.

Mass	Protein	Position	Sequence
830.4519	*β*-CN	192-198	AVPYPQR
2186.1678	*β*-CN	199-217	DMPIQAFLLYQEPVLGPVR
2909.5997	*β*-CN	199-224	DMPIQAFLLYQEPVLGPVRGPFPIIV
1759.9449	α_{S1}-CN	23-37	HQGLPQEVLNENLLR
847.5036	BSA	242-248	LSQKFPK
1001.5890	BSA	233-241	ALKAWSVAR
1153.5753	Trypsin	149-159	SSGTSYPDVLK
2163.0564	Trypsin	73-92	LGEDNINVVEGNEQFISASK

Table S3. Position, sequence and theoretical mass of corresponding peptides in tryptic digests of eel sample eluate.

Mr: monoisotopic mass; Delta: delta of experimental and calculated mass; M: missed cleavage;

[pT]: phosphorylation at threonine site; [pS]: phosphorylation at serine site; [Mo]: oxidation at methionine site.

**Position**	**Observed**	**Mr(expt)**	**Mr(calc)**	**Delta**	**M**	**Peptide Sequence**
6–9	506.9310	505.9237	505.2649	0.6588	0	K.FSPR.R
14–27	1765.4160	1764.4087	1764.7985	−0.3898	1	R.HV[pT]GSFESSFRFLR.E
28–37	1216.0040	1214.9967	1214.6255	0.3712	1	R.EEDADVLRIR.R
38–48	1311.0350	1310.0277	1310.5326	−0.5049	1	R.RG[pS]MSPEPENK.E
38–48	1327.0370	1326.0297	1326.5275	−0.4978	1	R.RG[pS][Mo]SPEPENK.E
54–60	1033.8560	1032.8487	1032.2912	0.5575	0	K.[Mo][pS]YEAEK.T
82–117	4474.7650	4473.7577	4474.7890	−1.0313	1	K.YEIPG[Mo]GDD[pS]VLEML[pS]YSKFSDLETWLCMPSTLLPR.S

Position	Observed	Mr(expt)	Mr(calc)	Delta	M	Peptide Sequence
101–117	2040.6830	2039.6757	2039.9696	−0.2939	0	K.FSDLETWLC[Mo]PSTLLPR.S
101–117	2104.6400	2103.6327	2103.9410	−0.3083	0	K.F[pS]DLETWLCMPSTLLPR.S
118–139	2356.7220	2355.7147	2356.0350	−0.3202	1	R.SRDSVCSLPPPSHENGTADTDR.E
120–139	2513.8450	2512.8377	2512.7335	0.1043	0	R.D[pS]VC[pS]LPPP[pS]HENG[pT]AD[pT]DR.E
140–157	2407.7910	2406.7837	2406.8354	−0.0517	0	R.E[pS]RPPEN[pT][pS]INL[pS]QCQER.K
140–158	2456.7530	2455.7457	2454.9641	0.7817	1	R.E[pS]RPPENT[pS]INL[pS]QCQERK.T
159–192	3824.4570	3823.4497	3823.8022	−0.3525	1	K.[pT]HLLPP[pS][pS]LMPVSASPTSSTPLRTTSTPLPKPNR.D
182–192	1531.2220	1530.2147	1530.5323	−0.3175	0	R.[pT][pT][pS][pT]PLPKPNR.D
229–239	1202.9880	1201.9807	1201.6302	0.3505	1	R.GETPSLSEVKR.F
243–257	1804.4070	1803.3997	1802.8799	0.5198	1	R.SVDSESLVPELWRDR.A

Position	Observed	Mr(expt)	Mr(calc)	Delta	M	Peptide Sequence
243–257	1884.5450	1883.5377	1882.8462	0.6915	1	R.[pS]VDSESLVPELWRDR.A
292–303	1431.1180	1430.1107	1430.6432	−0.5325	1	K.LRNIN[pS]LGA[pT]GR.Y
321–332	1436.1370	1435.1297	1435.7184	−0.5887	1	R.LNQRQ[pS]DAALIK.D
325–332	924.7180	923.7107	924.4317	−0.7210	0	R.Q[pS]DAALIK.D
333–371	4458.7720	4457.7647	4458.0985	−0.3338	1	K.DFRPLFLL[pS]GSAGSSQSLDRNFSIS[Mo]SQQ[Mo]QNLQLTQSK.K
353–371	2611.8510	2610.8437	2611.8932	−1.0495	0	R.NF[pS]I[pS]M[pS]QQMQNLQL[pT]Q[pS]K.K
507–517	1386.1490	1385.1417	1385.6122	−0.4705	1	R.RA[pS]ELSNQV[Mo]R.E
540–543	659.4270	658.4197	658.3203	0.0994	1	K.LYKR.M
562–587	2895.9990	2894.9917	2894.2463	0.7454	0	R.L[pS]V[pT]G[pS]TTLTVSFQEPASMNSAVVTK.Y
562–589	3266.2700	3265.2627	3265.3709	−0.1082	1	R.L[pS]V[pT]G[pS]TTLTVSFQEPASMNSAVVTKYK.V

Position	Observed	Mr(expt)	Mr(calc)	Delta	M	Peptide Sequence
597–614	2163.6100	2162.6027	2162.0313	0.5715	0	K.DF[pS]LLAGELILENLQ[pS]LK.C
637–655	2198.6080	2197.6007	2197.9235	−0.3228	0	K.GWGPAQL[pS]QPP[pS]AVPSNWK.D
666–673	974.6960	973.6887	973.4287	0.2600	0	R.GHIEA[Mo]ER.L
680–692	1722.3610	1721.3537	1721.4654	−0.1116	0	R.A[pT]HQHYCCGD[pT]SK.L
700–707	1143.9310	1142.9237	1143.4127	−0.4890	1	K.Q[pS]V[pS]R[pS]LK.H
705–715	1556.2810	1555.2737	1555.5874	−0.3137	1	R.[pS]LKHLFH[pS][pS]TK.F
716–721	802.5980	801.5907	800.4197	1.1710	1	K.FVK[pS]LK.R
734–766	3946.5450	3945.5377	3945.7795	−0.2418	0	K.DSLLVTNEDQIPIVEVDDSYSSSL[Mo]QDFLWFTK.L
734–766	4187.7630	4186.7557	4185.6785	1.0772	0	K.D[pS]LLV[pT]NEDQIPIVEVDD[pS]YSSSL[Mo]QDFLWFTK.L
767–775	1153.9040	1152.8967	1153.4896	−0.5929	0	K.LSCMWEDVR.W

Position	Observed	Mr(expt)	Mr(calc)	Delta	M	Peptide Sequence
823–834	1475.1720	1474.1647	1473.7453	0.4194	1	K.DRHGNVLLV[pT]VR.E
825–834	1123.8220	1122.8147	1122.6510	0.1638	0	R.HGNVLLVTVR.E
835–846	1677.2660	1676.2587	1676.5272	−0.2685	0	R.EMD[pS]LY[pS]FFNGK.W
835–852	2468.8850	2467.8777	2467.8908	−0.0131	1	R.E[Mo]D[pS]LY[pS]FFNGKWMQVSK.L
853–858	759.4820	758.4747	758.4399	0.0349	1	K.LQSQRK.S
901–909	976.7140	975.7067	975.5237	0.1831	0	K.LSSSVDQIK.V
927–933	986.7190	985.7117	985.3655	0.3463	0	R.DNWNV[pS]R.D
951–968	2068.5860	2067.5787	2067.8989	−0.3202	0	R.ADQA[pT]GCLLF[pS]ELQTAIK.S
969–982	1724.3350	1723.3277	1722.8818	0.4459	0	K.[pS]LLHQINLPLHQAK.H
1070–1081	1491.1620	1490.1547	1489.5027	0.6520	0	R.EAI[pT]D[pS]EV[pS]TAK.Q

Position	Observed	Mr(expt)	Mr(calc)	Delta	M	Peptide Sequence
1137–1158	2517.8510	2516.8437	2517.0479	−0.2042	1	K.NDSTSSNTDYVPTPSPSPEMRR.R
1137–1158	2534.7590	2533.7517	2533.0428	0.7089	1	K.NDSTSSNTDYVPTPSPSPE[Mo]RR.R
1137–1158	2613.8960	2612.8887	2613.0092	−0.1204	1	K.ND[pS]TSSNTDYVPTPSPSPE[Mo]RR.R
1245–1264	2480.8670	2479.8597	2480.0162	−0.1565	1	K.DLEDL[pS]L[pS]SYSVKTTDKPSR.S
1258–1264	805.5800	804.5727	803.4137	1.1590	0	K.TTDKPSR.S
1265–1274	1157.8130	1156.8057	1156.5529	0.2528	1	R.[pS]KFLADAPTK.R
1267–1274	942.7250	941.7177	941.4259	0.2918	0	K.FLADAP[pT]K.R
1277–1285	1241.0540	1240.0467	1240.5366	−0.4899	1	K.LL[pS]K[pS]HPQR.S
1281–1293	1797.4060	1796.3987	1796.6223	−0.2235	1	K.[pS]HPQR[pS]YFGGPHR.W
1297–1313	2386.7280	2385.7207	2385.7048	0.0159	0	R.VQ[pS]E[pS]H[pT]P[pS]L[pS]EGIYTR.Q

Position	Observed	Mr(expt)	Mr(calc)	Delta	M	Peptide Sequence
1354–1366	1457.1470	1456.1397	1455.6850	0.4547	1	R.IFVESCSKTSPCR.D
